# Automated Mobile Delivery of Financial Incentives for Smoking Cessation Among Socioeconomically Disadvantaged Adults: Feasibility Study

**DOI:** 10.2196/15960

**Published:** 2020-04-15

**Authors:** Darla E Kendzor, Michael S Businelle, Joseph J C Waring, Ashley J Mathews, Daryl W Geller, Jocelyn M Barton, Adam C Alexander, Emily T Hébert, Chaelin K Ra, Damon J Vidrine

**Affiliations:** 1 Oklahoma Tobacco Research Center Stephenson Cancer Center University of Oklahoma Health Sciences Center Oklahoma City, OK United States; 2 Moffitt Cancer Center Tampa, FL United States

**Keywords:** socioeconomic status, smoking cessation, incentives, mobile health, mobile phone

## Abstract

**Background:**

Socioeconomic disadvantage is associated with a reduced likelihood of smoking cessation. Smartphone ownership is increasing rapidly, including among low-income adults, and smartphone interventions for smoking cessation may increase access to smoking cessation treatment among socioeconomically disadvantaged adults.

**Objective:**

This study aimed to evaluate the feasibility of an automated smartphone-based approach to delivering financial incentives for smoking cessation.

**Methods:**

Socioeconomically disadvantaged adults initiating tobacco cessation treatment were followed from 1 week before a scheduled quit attempt through 26 weeks after the quit date. Participants received telephone counseling and nicotine replacement therapy. Smoking cessation was verified 5 times per week via smartphone prompts to self-report smoking status and submit a breath sample via a portable carbon monoxide (CO) monitor that was connected with participants’ smartphones. Identity was verified during smoking status assessments using smartphone-based facial recognition software. When smoking abstinence and identity were verified, an automated credit card payment was triggered. Participants were incentivized for abstinence on the quit date and up to five days per week during the first 4 weeks after the scheduled quit date, with additional incentives offered during postquit weeks 8 and 12. In total, participants had the opportunity to earn up to US $250 in abstinence-contingent incentives over the first 12 weeks of the quit attempt.

**Results:**

Participants (N=16) were predominantly female (12/16, 75%) and non-Hispanic white (11/16, 69%), black (4/16, 25%), or Hispanic of any race (1/16, 6%). Most participants (9/16, 56%) reported an annual household income of <US $11,000. During the first 4 weeks after the scheduled quit date, participants completed a median of 16 (out of 21; range 1-21) mobile smoking status assessments, and they earned a median of US $28 in abstinence-contingent incentives (out of a possible US $150; range US $0-US $135). Median earnings did not change during the 8- and 12-week incentivized follow-up periods (total median earnings over 12 weeks=US $28; range US $0-US $167). During the first 4 weeks after the scheduled quit date, participants abstained from smoking on a median of 5 (out of 21) assessment days (range 0-20). At the in-person follow-up visits, the expired CO-confirmed 7-day point prevalence abstinence rates were 19% (3/16) and 13% (2/16) at 12 and 26 weeks postquit, respectively. Overall, most participants reported that the system was easy to use and that they would recommend this treatment to their friends and family.

**Conclusions:**

Preliminary data suggest that this smartphone-based approach to verifying identity and smoking status and automating the delivery of abstinence-contingent incentives to a credit card is feasible for use among socioeconomically disadvantaged adults. However, continued refinement is warranted.

## Introduction

### Background

Smoking prevalence rates are disproportionately high among socioeconomically disadvantaged adults [1], and socioeconomic disadvantage is associated with a reduced likelihood of smoking cessation [2-7]. Although individuals of lower socioeconomic status (SES) are just as likely to initiate quit attempts, they are less likely to succeed than those of higher SES [8]. Similarly, smoking cessation interventions for low SES populations have produced very low abstinence rates at follow-up [9-12]. Treatment approaches are needed to target socioeconomically disadvantaged populations and to reduce barriers to access. Smartphone interventions potentially offer a means of increasing access to treatment across settings. Notably, smartphone ownership is increasing rapidly, even among low-income adults. According to the Pew Research Center, 81% of US adults overall and 71% of US adults with an annual household income of <US $30,000 reported that they owned a smartphone in 2019 [13]. Importantly, adults with an annual household income of <US $30,000 are more than 4 times as likely to rely solely on their smartphones to access the internet than those earning >US $75,000 annually [13]. Smartphone-based treatments may be used in conjunction with traditional empirically supported approaches (counseling and pharmacotherapy), while also incorporating innovative components such as real-time assessment and intervention.

Contingency management (CM), the tangible reinforcement of abstinence and other related outcomes, is highly effective for promoting drug and alcohol abstinence among individuals with substance use disorders [14-16]. In addition, there is accumulating evidence that CM is an effective approach for promoting smoking cessation in a variety of populations [17-33], including adults of lower SES [34-39]. Research suggests that financial incentives for smoking cessation may be particularly appealing among socioeconomically disadvantaged individuals [40,41]. The findings of two meta-analyses have indicated that financial incentives are associated with greater odds of behavior change for a variety of behaviors, particularly among lower SES individuals [42,43].

To date, financial incentive interventions for smoking cessation have primarily relied on in-person visits to verify smoking abstinence. However, internet [44-48] and mobile phone-based [49-51] CM approaches have been developed to reduce or eliminate the need for in-person visits. An internet-based approach has been evaluated in several studies [44-48], where participants access a study website and record themselves via Webcam as they provide breath samples using a loaned carbon monoxide (CO) monitor. Participants upload the recordings for staff review, and a monetary credit is applied to their study credit cards when abstinence and identity are verified. A similar approach has been employed in several studies, where mobile phones equipped with video cameras allowed participants to record themselves as they provided a CO breath sample and, then, uploaded the videos for staff review via a study website [49-51]. Previous mobile CM approaches have lacked automation and have required substantial effort from participants and staff to record and upload videos, verify abstinence and identity, and administer payments. Technologies such as SCRAM continuous alcohol monitoring [52] and Soberlink [53] have combined facial recognition software with alcohol breath tests to automate the process of identity verification, and this approach could also be applied to CO breath tests among individuals who are attempting to quit smoking.

### Objectives

The purpose of this project was to develop and test the feasibility of an automated smartphone-based CM approach that has the potential to allow socioeconomically disadvantaged adults to remotely benefit from smoking cessation treatments that offer financial incentives for evidence of smoking cessation. Feasibility was evaluated among socioeconomically disadvantaged males and females seeking smoking cessation treatment. This remote and automated intervention approach has the potential to increase the availability of financial incentive interventions for smoking cessation among individuals who are unable to attend in-person visits while reducing the need for staff monitoring and manual payment disbursement. This study represents a first step toward establishing the feasibility of a mobile CM intervention approach that is designed to be highly scalable and to ultimately facilitate the widespread adoption of CM treatments for smoking cessation.

## Methods

### Mobile Contingency Management

The INSIGHT mobile health (mHealth) platform is a versatile interface that empowers researchers to build, test, and launch smartphone-based assessments and interventions. With this platform, investigators have the ability to select how they would like their assessments or interventions deployed (eg, assessment type and interval, algorithms for delivering treatment content) [54]. Researchers may also enter their preferred assessment and intervention content (eg, surveys, treatment messages, videos). The following technologies were newly integrated into the INSIGHT platform for this study: (1) the Bedfont iCO Smokerlyzer CO monitor that connects with mobile phones [55] to remotely verify smoking abstinence, (2) Microsoft facial recognition software (Azure Face application programming interface [API]) [56] to compare the participant’s face at the time of the breath sample submission with a photo taken at baseline for identity verification, and (3) reloadable Greenphire ClinCards (ie, credit card) [57] for the remote delivery of abstinence-contingent incentives that are automatically triggered by biochemical confirmation of self-reported smoking abstinence (via smartphone-based ecological momentary assessment [EMA]). This newly developed intervention approach was tested for feasibility among socioeconomically disadvantaged adults.

### Participants

Individuals were screened for eligibility following referral to the Tobacco Treatment Research Program (TTRP), which is located on the University of Oklahoma Health Sciences Center campus in Oklahoma City. The TTRP offers free tobacco cessation counseling and pharmacotherapy to the public, while also facilitating the recruitment, screening, and enrollment of participants into research studies [58]. TTRP referrals are primarily received through the electronic medical record, and they are also received via phone, internet, fax, and word-of-mouth. Following referral, participants are screened for ongoing studies at the TTRP over the phone and scheduled for an initial in-person treatment appointment. Most of the screening criteria for this study were assessed over the phone and verified in person, with the exception that CO level and literacy were only assessed in person. Interested participants were eligible for the study if they (1) were uninsured or received Medicaid benefits, (2) earned a score ≥4 on the Rapid Estimate of Adult Literacy in Medicine [59] indicating >6th grade English literacy level, (3) were willing to quit smoking 7 days from their first visit, (4) were 18 to 65 years of age, (5) had a CO level of ≥8 parts per million (ppm) suggestive of current smoking, and (6) were smoking ≥5 cigarettes per day. Participants were excluded from the study if they reported that they were (1) pregnant or breastfeeding, (2) had uncontrolled hypertension, (3) had a myocardial infarction within the past 2 weeks, (4) had an allergy to adhesive tape, or (5) were unwilling to use nicotine replacement therapy (NRT). Informed consent was obtained from eligible participants.

Participants referred to the TTRP who met eligibility criteria over the phone were scheduled for an in-person screening and enrollment visit (n=188). Of those, 91 attended their scheduled screening appointment, and 59 were eligible for this study. Of the 59 eligible patients, 36 were enrolled in another ongoing, higher priority study, 20 were enrolled in this study, and 3 declined to participate in either study. Of the 32 who were not eligible to participate, the reasons for exclusion were (in order of frequency): CO <8 ppm (n=10), reading level <6th grade (n=8), currently insured (besides Medicaid, n=6), smoking <5 cigarettes per day (n=3), uncontrolled hypertension (self-reported, n=3), unwilling to use NRT (n=2), or they were not ready to quit smoking in the next 7 days (n=1). Note that more than one reason for exclusion was possible. Those who were not eligible for the study were offered standard TTRP treatment. Participants (n=20) were enrolled in the study between August 2018 and February 2019, and final follow-up visits were completed in August 2019. Participants were intentionally enrolled slowly to ensure that the technology was working properly and to resolve technical problems as they arose before enrolling additional participants.

### Measures

Smoking status was assessed on the scheduled quit day and on 5 days each week for 4 weeks following the scheduled quit date via self-report and CO levels with the portable Bedfont iCO Smokerlyzer (connected with a smartphone). Self-reported abstinence from smoking since 10 PM the previous evening (reported via smartphone-based EMAs) was verified with a CO level of <10 ppm on the quit day (given the recency of quitting). Consistent with current recommendations [60], a more stringent CO level of <6 ppm was required to corroborate smoking abstinence on all subsequent days. Key feasibility metrics included the number of smoking status assessments completed, number of facial recognition assessments initiated and working as expected, number of days of biochemically verified abstinence, abstinence-contingent incentives earned, counseling sessions completed, weeks of NRT requested/received, participant perceptions of the interventions, and attendance at follow-up visits. Participants were scheduled for in-person follow-up visits at 12 and 26 weeks post the quit date, where they were asked if they had smoked in the past 7 days. Participants’ CO was measured at in-person visits with a Vitalograph BreathCO monitor, and a CO level of <6 ppm provided biochemical confirmation of abstinence. Note that participants’ CO values and the CO thresholds used to verify abstinence were not shared with participants.

### Procedure

#### Treatment

All participants were provided with the recommended components of an intensive tobacco treatment intervention [61]. Specifically, standard treatment included: (1) an in-person counseling session 1 week before the scheduled quit date with a certified tobacco treatment specialist (CTTS), (2) 5 weekly telephone counseling sessions with a CTTS starting on the scheduled quit date, (3) a 2-week supply of NRT (patches and gum or lozenges) during the first session, and (4) additional mailed patches and gum as needed for 12 weeks. The counseling sessions covered the following topics: (1) the health benefits of quitting, (2) mood/stress management strategies, (3) making positive lifestyle changes, (4) coping skills, and (5) relapse prevention. The CTTS checked in with participants each week about the difficulties and successes they experienced related to their quit attempt, and they planned for any challenging situations that were anticipated.

#### Paid Assessments

Participants earned US $30 to complete the in-person baseline visit that included Web-based questionnaires delivered via the research electronic data capture software platform (REDCap) [62,63], US $30 per week from the quit date through 4 weeks postquit for completing Web-based assessments via REDCap, US $40 for completing a Web-based REDCap assessment at 8 weeks postquit, US $40 for completing a 12-week postquit in-person visit (including Web-based questionnaires via REDCap and smoking status assessment), and US $20 for completing a 26-week in-person follow-up visit (including Web-based REDCap questionnaires and smoking status assessment). Participants also earned up to US $150 depending on the percentage of prompted smartphone-based EMAs they had completed (including smartphone-based smoking status assessments). Note that questionnaire and EMA data not directly related to the primary purpose of the study are not presented or discussed further here. Overall, participants had the possibility of earning up to US $430 for the completion of questionnaires and EMAs (that were not contingent on abstinence), and all payments were credited to participants’ ClinCards.

#### Smartphone-Based Smoking Status Assessment

Participants were provided with a Smokerlyzer iCO monitor, a Samsung Galaxy S7 smartphone preloaded with the INSIGHT app, and a remotely reloadable Greenphire ClinCard (credit card). Participants were offered unlimited calling and 2 gigabytes of data for personal use along with the phones. Participants were prompted via ring/vibration and a pop-up window (delivered through the INSIGHT app), indicating that it was time to complete a smoking status assessment (including a CO breath sample). Smoking status assessments were conducted at the end of the day (2 hours before typical bedtime) on the scheduled quit day and 5 days out of each week during the first 4 weeks after the quit day (days were selected to appear random). Participants received up to 2 additional reminder prompts (5 min apart) if the first and second assessment prompts were snoozed. Thus, they had a total of 10 min to complete the CO sample submission. If they missed the first assessment entirely (ie, did not snooze), they were prompted 30 min later, and if they missed the second assessment entirely, then 30 min later they were prompted a third time. After the third missed prompt, participants were no longer able to complete the smoking status assessment. In addition, participants were prompted to complete 5 smoking status assessments during weeks 8 and 12.

While participants provided a breath sample, 2 photos were taken at random times during the 20-second exhalation period using the front-facing smartphone camera. It was not apparent to participants exactly when the phone was taking pictures; rather, participants were only aware that during the process of providing the breath sample, they would be photographed for identity confirmation. The smartphone app compared the photos in vivo with a photo taken at each participant’s baseline appointment to verify identity. Facial recognition assessments were considered to have worked as expected when identity was accurately processed as either a match or a nonmatch with the baseline photo. Study staff reviewed all of the stored photos, and nonmatches in which participant identity was confirmed by research staff were considered to be failures. There were no instances of erroneous matches.

#### Abstinence-Contingent Incentives

Abstinence-contingent incentives were automatically delivered to the credit card based on daily self-reports of smoking abstinence and CO levels. This was accomplished through an API that allowed the INSIGHT app to communicate with Greenphire. A spreadsheet was automatically generated and sent to Greenphire, which contained the payment amounts owed to participants’ ClinCard accounts. These payments were automatically drawn and disbursed from an account with Greenphire that was set up in advance of the study. On the scheduled quit day, participants who were biochemically confirmed abstinent from smoking since 10 PM the previous evening received a US $20 credit on their ClinCard (incentive schedule is detailed in Table 1). After the quit day, a payment was earned following a smartphone-based self-report of abstinence during the past 24 hours combined with a breath CO sample of <6 ppm and a facial recognition match. For each abstinent day during the first week postquit, a US $4 credit was earned. The incentive amount per abstinent day increased by US $1 with each week of consecutive abstinence until 4 weeks postquit, when continuously abstinent participants earned US $7 per abstinent day. Participants who provided 5 negative breath samples within a week additionally received a US $5 bonus through 4 weeks postquit. Participants who were nonabstinent (or who did not provide a sample) did not earn incentives that day but could begin earning incentives for abstinence again on their next abstinent day, although the amount was reset to the starting level of US $4 per abstinent day. Participants earned US $8 per abstinent day during weeks 8 and 12 postquit, with a US $10 bonus for 5 negative samples each week. Payments did not reset or escalate during the 8- and 12-week follow-up periods. Altogether, participants could earn up to a possible US $250 for biochemically verified abstinence. The incentive schedule (Table 1) was adapted from the schedules utilized with socioeconomically disadvantaged adults in previous research by the investigators [34,36].

**Table 1:** Incentive schedule.

Weeks postquitting	Abstinence-contingent incentives^a^ (US $)	Total^b^ (US $)
Quit day	20 for negative CO^c^ sample	20
Week 1	4 per negative CO sample (up to 20 + 5 bonus)	25
Week 2	5 per negative CO sample (up to 25 + 5 bonus)	30
Week 3	6 per negative CO sample (up to 30 + 5 bonus)	35
Week 4	7 per negative CO sample (up to 35 + 5 bonus)	40
Week 8	8 per negative CO sample (up to 40 + 10 bonus)	50
Week 12	8 per negative CO sample (up to 40 + 10 bonus)	50

^a^Participants earned a bonus incentive of US $5 when they achieved biochemically verified abstinence on all 5 smoking status assessments during the first 4 weeks after the scheduled quit date. The bonus payment increased to US $10 during postquit weeks 8 and 12.

^b^Partcipants could earn up to US $250 in abstinence-contingent incentives over the 12-week intervention period.

^c^CO: carbon monoxide.

#### Problem Reporting and Resolution

Participants were able to report *bugs* or contact staff with questions about using the smartphone by clicking *call study staff* on the main menu of the app. In addition, participants were asked if they had experienced any problems with the app as part of the end-of-day assessments during the first 4 weeks postquit. If they said yes, they were prompted to offer detailed information in an open text box. Participants also had weekly scheduled counseling calls where they could ask questions and report problems. Over the course of the study, instructions to participants about how to successfully use the facial recognition component of the app were improved. We began offering more detailed information (training and paper handout) about optimal facial placement on the phone screen when providing a CO breath sample, and we emphasized the importance of maintaining consistency in appearance related to eyeglasses, hairstyles, background, and facial position. Proper facial placement was important because the app did not provide feedback to the participants during facial recognition assessments (this will be addressed in future versions of the app). In addition, the mHealth programming staff worked through several technical problems that arose during the study, which interfered with the proper functioning of the facial recognition component of the app. Owing to these early improvements, outcomes were characterized separately for the first and second halves of participants enrolled to demonstrate how these improvements might have impacted study outcomes (see the Analytic Plan section).

#### Analytic Plan

Descriptive statistics were generated for key feasibility metrics (noted above in the measures section), including medians and ranges for continuous variables and frequencies for categorical variables. The sample was also divided into the first and second halves enrolled, and sample descriptives were generated for each half to illustrate the impact of improvements to the protocol and smartphone app. Nonparametric Mann-Whitney *U* tests and chi-square analyses were conducted to compare the first and second halves of participants enrolled on key metrics. Statistical comparisons may provide information about trends toward improvement, despite the small sample size.

## Results

### Participant Characteristics

Of those enrolled, 4 participants did not complete any smoking status assessments on their smartphones. Because these participants did not initiate the financial incentives component of the intervention, they were excluded from the primary analyses. The remaining 16 enrolled participants were predominantly female (12/16, 75%), with a median age of 47 years (range 18-63 years). Participants were 69% (11/16) non-Hispanic white, 25% (4/16) non-Hispanic black, and 6% (1/16) Latino/Hispanic. Participants reported a median of 12.5 years of education (range 9-14 years), with 25% (4/16) completing less than 12 years of education. Before quitting, participants reported smoking a median of 19.0 (range 5-40) cigarettes per day for 30.5 years (range 6-49). Most participants were not employed (12/16, 75%), and most reported an annual household income of <US $11,000 (9/16, 56%). Of the 16 individuals who participated in the intervention, only 1 phone was not returned (1/16, 6%). However, 3 of the 4 individuals who did not participate in the smartphone-based financial incentives component of the intervention did not return their phones. Thus, 4 phones were not returned among the 20 enrolled participants overall (4/20, 20% phone loss).

Although statistical comparisons between enrolled participants who initiated (n=16) or did not initiate (n=4) the intervention were not feasible given the small sample size, it is worth noting that those who did not initiate the intervention appeared to differ from those who initiated the intervention on a variety of characteristics. Those who did not initiate the intervention were less likely to be female (2/4, 50% vs 12/16, 75%), and were more likely to report being black (2/4, 50% vs 4/16, 25%) or American Indian (1/4, 25% vs 0/16, 0%). In addition, those who did not initiate the intervention were younger (median 41.5; range 36-45 years vs 47.0 years, range 18-63 years), smoked fewer cigarettes per day (median 12.5; range 5-20 vs 19.0; range 5-40), smoked for fewer years (median 20.0; range 13-30 years vs 30.5; range 6-49 years), had less education (median 10.5; range 9-12 years vs 12.5; range 9-14 years), and reported less income (4/4, 100% vs 9/16, 56% with <US $11,000 in annual household income).

### Completion of Mobile Smoking Status Assessments

Participants completed a median of 16 (range 1-21) of the 21 possible iCO/smoking status assessments during the first 4 weeks postquit (ie, 1 on the quit date, and 5 per week thereafter). Notably, the final 8 participants enrolled had higher median completion rates relative to the first 8 enrolled (19.5 completed assessments, range 14-21 vs 10 completed assessments, range 1-20; *P*=.02). Unfortunately, smoking status assessment completion rates declined during week 8 (median 3.0 completed assessments out of a possible 5) and declined even further during week 12 (median 1.5 assessments completed out of a possible 5; Table 2).

**Table 2:** Improvements in treatment-related variables for the first to second half of the participants enrolled (N=16).

Postquit date and treatment-related variables		All participants	First half enrolled	Last half enrolled	*P* value^a,b^
**Weeks 1-4, median (range)**					
	Days carbon monoxide-confirmed abstinent^c^ (out of 21)	5.0 (0.0-20.0)	3.0 (0.0-11.0)	8.5 (1.0-20.0)	.07
	Completed smoking status assessments^c^ (out of 21)	16.0 (1.0-21.0)	10.0 (1.0-20.0)	19.5 (14.0-21.0)	.02
	Telephone counseling sessions completed (out of 5)	5.0 (1.0-5.0)	4.5 (1.0-5.0)	5.0 (4.0-5.0)	.20
	Abstinence-contingent incentives earned (US $; up to US $150)	28.0 (0.0-135.0)	20.0 (0.0-72.0)	60.0 (16.0-167.0)	.05
**Week 8, median (range)**					
	Days carbon monoxide-confirmed abstinent^d^ (out of 5)	0.0 (0.0-4.0)	0.0 (0.0-1.0)	1.0 (0.0-4.0)	.13
	Completed smoking status assessments^d^ (out of 5)	3.0 (0.0-5.0)	2.0 (0.0-5.0)	4.0 (0.0-5.0)	.16
	Abstinence-contingent incentives earned (US $; up to US $50)	0.0 (0.0-24.0)	0.0 (0.0-8.0)	4.0 (0.0-24.0)	.28
**Week 12**					
	Days carbon monoxide-confirmed abstinent^d^ (out of 5), median (range)	0.0 (0.0-3.0)	0.0 (0.0-1.0)	0.0 (0.0-3.0)	.65
	Completed smoking status assessments^d^ (out of 5), median (range)	1.50 (0.0-4.0)	1.50 (0.0-3.0)	1.50 (0.0-4.0)	.57
	Weeks of NRT^e^ (out of 12), median (range)	10.0 (2.0-12.0)	10.0 (2.0-12.0)	10.0 (2.0-12.0)	.86
	Abstinence-contingent incentives earned (US $; up to US $50), median (range)	0.0 (0.0-16.0)	0.0 (0.0-16.0)	0.0 (0.0-16.0)	.51
	Attended in-person follow-up visit, n (%)	13.0 (81.0)	6.0 (75.0)	7.0 (88.0)	.52
	Carbon monoxide-confirmed 7-day point prevalence abstinence, n (%)	3.0 (19.0)	1.0 (13.0)	2.0 (25.0)	.52
**Week 26**					
	Attended in-person follow-up visit, n (%)	11.0 (69.0)	5.0 (63.0)	6.0 (75.0)	.59
	Carbon monoxide-confirmed 7-day point prevalence abstinence, n (%)	2.0 (13.0)	0.0 (0.0)	2.0 (25.0)	.13

^a^Mann-Whitney *U* tests were conducted for continuous variables, and chi-square analyses were conducted for dichotomous variables.

^b^*P* values reflect the difference between the first 8 participants and the last 8 participants enrolled in the study.

^c^Self-reported and carbon monoxide–confirmed abstinence were assessed on the day after quitting and on 5 days per week for 4 weeks after the scheduled quit attempt (21 total assessments).

^d^Smoking status was additionally assessed 5 times per week during weeks 8 and 12 postquit date. Assessments where participants self-reported smoking but did not complete the iCO assessment were not considered missing.

^e^NRT: nicotine replacement therapy.

### Facial Recognition

Over the 12 week study period, there were 31 possible facial recognition assessments per participant (ie, 496 total possible for all 16 participants). Overall, the 16 study participants collectively initiated 282 facial recognition assessments as part of the smoking status assessments, and 48.6% (137/282) of those assessments worked as expected. Among the first 8 participants, 127 facial recognition assessments were initiated, and 30.7% (39/127) of those assessments *worked as expected*. Among the last 8 participants, 155 facial recognition assessments were initiated, of which 98 (63.2%) worked as expected. No instances were noted where someone other than the participant was photographed during a smoking status assessment. Although we do not have detailed documentation of the reasons for every instance of facial recognition failure, the failures seemed to fall into 3 broad categories: (1) improper facial placement in the frame during smoking status assessments (eg, face partially in the photo), (2) inconsistent appearance between the baseline photo and the photos taken during smoking status assessments (eg, inconsistencies in wear of hairstyles, glasses, hats), and (3) technical problems or *bugs*. The former categories were addressed by providing participants with additional guidance about proper facial placement and the importance of consistency in appearance across assessments. The latter technical problems were addressed by the programming staff as they arose.

### Treatment Engagement

All participants completed the in-person, prequit counseling session. After the initial visit, participants completed a median of 5 out of 5 possible weekly telephone counseling sessions (Table 2). A total of 69% (11/16) of participants completed all 5 counseling calls, whereas 19% (3/16) completed 4 counseling calls, 6% (1/16) completed 2 calls, and 6% (1/16) completed 1 call. A 2-week supply of NRT was offered at the in-person visit, and participants could request an additional 2-seek supply every 2 weeks for up to 12 weeks. Additional requested NRT was mailed to the participants. A 2-week supply included 14 patches and a box of 2 mg or 4 mg of nicotine gum or lozenges, depending on participant preference and smoking level (each box contained 100 pieces of gum or 108 lozenges). Participants requested and received a median of 10 weeks of NRT (range 2-12 weeks), with 13% (2/16) receiving a 2 week supply, 13% (2/16) receiving 4 weeks, 19% (3/16) receiving 3 weeks, 25% (4/16) receiving 10 weeks, and 31% (5/16) receiving a 12-week supply.

### Perceptions of the Intervention

Of the 16 study participants, 14 (88%) completed the Web-based intervention perceptions questionnaire via REDCap 4 weeks after the scheduled quit date. Note that the 2 participants with missing data were among the first half of the participants enrolled in the study. Overall, most participants found the intervention to be easy to use and helpful (for details, see Table 3). In addition, participants reported that they had problems with the app: “never” (4/16, 25%), “once or twice” (4/16, 25%), “a few times” (4/16, 25%), “several times” (1/16, 6%), and “daily or every other day” (1/16, 6%). Participants reported that the smartphone app and smoking monitor were correct in determining whether or not they were smoking: “never” (2/16, 13%), “some of the time” (2/16, 13%), “about half of the time” (1/16, 6%), “most of the time” (4/16, 25%) and “always” (5/16, 31%). Participants reported that it was difficult to find the iCO monitor when they needed to complete a smoking assessment: “never” (8/16, 50%), “a few times” (2/16, 13%), and “several times” (4/16, 25%). Finally, in response to the statement “earning financial incentives for quitting helped me to quit again after I had smoked” participants chose “I never smoked/lapsed after I quit” (3/16, 19%), “disagree” (1/16, 6%), “neither agree nor disagree” (5/16, 31%), “agree” (3/16, 19%), and “strongly agree” (2/16, 1%).

**Table 3:** Participants’ perceptions of the automated mobile contingency management intervention 4 weeks after a scheduled quit attempt (N=16).

Perceptions^a^	Agree or strongly agree, n (%)	Neither agree or disagree, n (%)	Disagree or strongly disagree, n (%)
The smartphone app was easy to use overall	12 (75)	2 (13)	0 (0)
My overall opinion of the smartphone app was positive	11 (69)	2 (13)	1 (6)
The smoking monitor was easy to use	11 (69)	1 (6)	2 (13)
It was difficult to blow into the smoking monitor while keeping my face in front of the smartphone screen	4 (25)	3 (19)	7 (44)
It was easy to tell how much I had earned for quitting each day/week by checking the payment screen	12 (75)	1 (6)	1 (6)
The opportunity to earn financial incentives for quitting helped keep my motivation for quitting high	11 (69)	3 (19)	0 (0)
Earning financial incentives for quitting helped me to feel more confident in my ability to quit	11 (69)	3 (19)	0 (0)
Earning financial incentives for quitting smoking helped me to successfully quit smoking	5 (31)	7 (44)	2 (13)

^a^A total of 88% (14/16) participants completed the perception survey 4 weeks after the scheduled quit date. As a result, the frequencies across the rows do not add up to 100%. The omitted 13% reflects the missing responses of 2 participants who were among the first half of the participants enrolled.

### Smoking Abstinence and Incentives Earned

See Table 2 for details about smoking abstinence and incentive earnings across the study visits. Participants were biochemically confirmed abstinent on a median of 5 days (range 0-20 days) out of 21 assessment days during the first 4 weeks postquit, with the last 8 participants achieving more abstinent days relative to the first 8 enrolled (median 8.5 abstinent days, range 1-20 vs 3 abstinent days, range 0-11; *P*=.07). Owing to low completion rates, evaluations of smoking status via smartphone assessments during weeks 8 and 12 were problematic. Over the entire 12-week incentive period, participants earned a median of US $28 (range US $0-US $167, out of US $250 possible) in abstinence-contingent incentives. The average earnings were greater among the last 8 participants enrolled than the first 8 participants (median US $60, range US $16-US $167 vs $20, range US $0-US $72; *P*=.05).

Participants were asked to attend 2 in-person follow-up visits at 12 and 26 weeks after the scheduled quit date to assess smoking status. Self-reported, biochemically confirmed (CO breath sample <6 ppm) 7-day point prevalence abstinence rates were 19% (3/16) and 13% (2/16), respectively, at the 12- and 26-week in-person follow-up visits. Attendance rates at the in-person follow-up visits were 81% (13/16) and 69% (11/16), respectively, at 12 and 26 weeks postquitting date, with those who did not attend considered to be smoking. As with the smartphone metrics, abstinence and attendance rates at in-person visits were higher among the latter half of participants enrolled relative to the first half of participants, suggesting that the delivery of the intervention may have improved over time (Table 2).

## Discussion

### Principal Findings

Overall, preliminary data suggest that this remote approach to verifying smoking status and participant identity and automating the delivery of abstinence-contingent incentives to a credit card is feasible for use with socioeconomically disadvantaged adults seeking smoking cessation treatment. During the study, improvements made to the protocol and smartphone app corresponded with improvements on most study metrics between the first half and the latter half of participants (Table 2) including (1) smoking status assessments completed, (2) facial recognition assessments *working as expected*, (3) telephone counseling sessions completed, (4) biochemically verified abstinent days, and (5) incentives earned for abstinence. Notably, most participants reported that they would recommend this intervention to their friends and family. At the in-person follow-up visits, CO-confirmed 7-day point prevalence abstinence rates were 19% (3/16) and 13% (2/16) at 12 and 26 weeks postquit date, respectively, which was high relative to other studies with socioeconomically disadvantaged individuals [9-12]. Attendance at follow-up visits was good, with in-person follow-up rates of 81% (13/16) and 69% (11/16) at 12 and 26 weeks postquit date, respectively.

### Opportunities for Improvement

Despite positive indicators of overall feasibility, there is still much opportunity for improvement. In total, 4 out of 20 participants enrolled (4/20, 20%) did not initiate engagement with the app, and therefore did not earn abstinence-contingent financial incentives. Approaches will be needed in future research to monitor lack of engagement and automate assistance early in the intervention as needed. For example, participant app use could be monitored, possibly through automated notifications to staff, to prompt staff outreach with the goal of increasing initial engagement for those who need it. It is possible that participants who did not engage with the app were less comfortable utilizing the technology, had experienced a problem that they were unable to resolve, or simply needed a reminder or encouragement to initiate the intervention. Although all participants were provided with phones for this study, 2 of the 4 participants who did not initiate the app component of the intervention reported that they did not own a mobile phone at the time they enrolled in the study. In contrast, among the 16 participants who engaged with the app, only 1 participant reported that he/she did not own a mobile phone.

Notably, although the mobile smoking status assessment completion rate was very high during the first 4 weeks postquit among the latter half of participants (ie, median of 19.5 out of 21 completed assessments), assessment completion rates declined substantially over time. The completion of these assessments is crucial to this type of intervention; therefore, a continued focus on improving compliance is warranted. It is possible that, on occasion, participants were unprepared for smoking status assessments because they were prompted by the app at inconvenient times. An alternative approach used by Dallery et al [44,48] requires that participants self-initiate CO breath samples twice per day at least eight hours apart. This strategy could maximize convenience for participants because they choose their assessment times, thus reducing missed assessments, while also limiting participants’ ability to smoke without detection. The percentage of completed smoking status assessments decreased dramatically between the first 4 weeks and the 8- and 12-week assessments, suggesting that participants became disengaged with the app during the *break* periods. These follow-up assessment/incentive weeks could be eliminated and/or the continuous incentive period could be extended to 6 weeks to increase continuous engagement with the app and maximize the influence of the intervention. Alternatively, smartphone assessments and/or messaging could be added between the incentive weeks to encourage sustained participant engagement.

### Challenges Associated With Facial Recognition

Incorporating facial recognition software posed challenges. There were several technical *bugs* that interfered with proper facial recognition processing. This had the effect of preventing incentive payments for participants and subsequently required attention from study staff to manually pay participants. During internal testing, we found that photos could be used to pass the identity verification component of the smoking status assessments. However, this deception becomes obvious when the stored photos are reviewed, and to our knowledge, this did not occur in this feasibility study. A random review of stored photos will be incorporated in future studies to identify attempted deceptions and other problems. Future iterations of the smartphone intervention will benefit from the resolution of problems in this study, and presumably, facial recognition metrics will continue to improve in future studies.

It is noteworthy that a recent evaluation of facial recognition software uncovered bias in facial recognition systems; for example, greater false match rates have been identified among women than men, and among African Americans than Caucasians [64]. Furthermore, false positives are more likely to occur when comparing images of individuals of the same sex, age, and ethnicity. Current recommendations to mitigate these problems include the use of training data that are diverse and globally derived, assessment of both the face and the iris, and modification of matching thresholds based on demographics [64]. As facial recognition software developers begin following these recommendations, the accuracy of the software may be expected to improve.

### Limitations

This study has limitations. First, the sample size was small, and the study did not include a comparison group, thus limiting the ability to draw conclusions about intervention effectiveness. Nevertheless, the sample size and design are appropriate for the initial feasibility testing, which will inform improvements to future versions of the intervention. Regarding biochemical verification of abstinence, it is possible that participants could have smoked between assessments given the short half-life of CO. However, submitting negative CO samples on a near-daily basis while continuing to smoke would have required participant knowledge of CO half-life in combination with discipline in the timing of their daily smoking. For these reasons, it seems unlikely that smoking would go undetected for an extended period of time. Notably, the current intervention includes more frequent smoking status assessments than many other intervention studies utilizing incentives, which have included biochemical verification at weekly intervals or at key follow-up points [34,37,38]. Cotinine assessment provides a longer window of smoking detection, and could be included in future studies of mobile CM when in-person follow-up assessments are part of the protocol (ie, identity could not be easily verified if cotinine was assessed remotely).

Another potential concern about remote biochemical verification of abstinence relates to the limited ability to verify if a participant is actually exhaling through the iCO monitor. The iCO monitor must be plugged-in during the smoking status assessment; otherwise, the app records an error code, and the assessment cannot be completed. In addition, second-to-second variability in CO readings during the exhalation period (20 seconds) can be assessed and is currently being explored as a means of verifying exhalation. Specifically, the variability in CO is expected to be greater during the exhalation period than during the period before exhalation (ie, when participants are instructed to hold their breath) even among those who are not smoking.

Notably, participants were provided with smartphones in this feasibility study to increase consistency and control over app functionality. In future testing, participants’ own phones will be used to deliver the intervention to verify usability across multiple types of phones and smartphone service plans. Similarly, in-person visits will be eliminated in future iterations of this research, in favor of completely remote smartphone setup, assessment, and treatment to demonstrate the scalability of the intervention.

### Costs and Potential for Real-World Utility

If this intervention is ultimately shown to be efficacious in a fully powered trial, this automated CM approach has numerous potential applications in settings where smokers may have limited access to smoking cessation resources or transportation. Plausibly, mobile CM could be paired with the services offered through state tobacco cessation quitlines and health care clinics to increase the reach of incentives-based interventions to socioeconomically disadvantaged individuals. For example, Mundt et al [39] demonstrated that incentivizing both quitline counseling calls and biochemically verified smoking cessation among Medicaid recipients improved cessation rates relative to a nonincentivized control group and the costs of the intervention compared favorably with other treatments. Nevertheless, real-world factors may impact the feasibility of implementation in different settings, including the availability of funding for the intervention and operational factors unique to each setting.

Notably, the incentive schedule utilized in this study and the investigators’ previous work [34,36] is low-cost and potentially cost-effective and compares favorably with the cost of other empirically supported and commonly used tobacco cessation treatments (eg, 10 weeks of generic nicotine patch costs approximately US $130). Previously, we found that cessation rates among socioeconomically disadvantaged adults incentivized for smoking cessation were more than double those of the standard treatment only control group for an additional cost of only US $63 (on average) per participant [34]. Cost-effectiveness research is needed to evaluate the practicality of this approach, and it is necessary to evaluate CM interventions more broadly. The optimal magnitude of abstinence-contingent incentives and the length of time that incentives should be offered must be explored.

Future research will focus on continued refinement and evaluation of this automated mobile CM approach to smoking cessation among socioeconomically disadvantaged individuals. Effective interventions with the potential for broad reach are especially important in places such as Oklahoma where nonmetropolitan residence is common, and rates of poverty and lack of health insurance are elevated. Once refined and tested in a fully powered trial, this fully automated CM approach to smoking cessation has the potential to facilitate intervention delivery to socioeconomically disadvantaged individuals across settings and locations.

## Abbreviations

API: application programming interface
CM: contingency management
CO: carbon monoxide
CTTS: certified tobacco treatment specialist
EMA: ecological momentary assessment
mHealth: mobile health
NCI: National Cancer Institute
NRT: nicotine replacement therapy
ppm: parts per million
SES: socioeconomic status
TTRP: Tobacco Treatment Research Program
